# Genetic Risks to Nicotine Dependence Predict Negative Mood and Affect in Current Non-Smokers

**DOI:** 10.1038/srep09521

**Published:** 2015-03-31

**Authors:** Xiangning Chen, Steven H. Aggen, Jingchun Chen, Lingxi Li, Kenneth S. Kendler, Melissa Blank, Thomas Eissenberg

**Affiliations:** 1 Virginia Institute for Psychiatric and Behavioral Genetics, Virginia Commonwealth University; 2 Department of Human and Molecular Genetics, Virginia Commonwealth University; 3 Department of Psychology, West Virginia University; 4 Department of Psychology and Center for the Study of Tobacco Products, Virginia Commonwealth University

## Abstract

Nicotine is the psychoactive agent involved in nicotine dependence. However, nicotine as a drug, and its effects on human psychology are largely under-investigated in genetic studies. In this study, we recruited 208 current non-smokers to evaluate the effect of nicotine and its relationship to genetic risks to nicotine dependence. Exploratory and confirmatory factor analyses, as well as measurement invariance testing, were conducted to evaluate the latent factor structures of the POMS, PANAS and DEN questionnaires across 3 nicotine doses. Structural models were used to examine the effects of nicotine and their relationship to genetic risks of nicotine dependence. We found that nicotine administration led to the change of both measurement construct and factor means, indicating the causal effect of nicotine on the psychological responses. The genotypes of rs588765 predicted the scores of the DEN Confused and Dizzy factors (p = 0.0003 and 0.001 respectively), and rs16969968 and rs588765 were associated with the PANAS Nervous factor (p = 0.006 and 0.007 respectively). Our study suggested that genetic risk of nicotine dependence is associated with acute psychological responses. The integration of psychometric analyses and dose effects could be a powerful approach for genetic study of nicotine dependence.

In the last several years, genetic studies of smoking and nicotine dependence have made significant progress, exemplified by the identification of the CHRNA5-CHRNA3-CHRNB4 locus. Genetic variants at this locus have been shown to be associated with a number of smoking phenotypes such as the number of cigarettes smoked per day^1^,^2^,^3^,^4^, nicotine dependence as defined by DSM IV^5^,^6^ and the Fagerström Test for Nicotine Dependence^7^,^8^, age of smoking onset^5^,^9^,^10^ and plasma cotinine concentration^11^. This locus includes the CHRNA5, CHRNA3 and CHRNB4 genes. While a non-synonymous SNP, rs16969968, in the CHRNA5 gene has emerged as a functional candidate, it may not be the one tagging the strongest association signal^12^. Further analyses reveal that there are multiple statistically independent association signals at this locus, represented by SNPs rs16969968, rs588765 and rs578776^13^.

Nicotine, the primary psychoactive substance in cigarette smoke, has broad psychological effects on humans^14^. When first exposed to nicotine, either by smoking or other routes of administration, some people feel pleasant and relaxed, while others feel dizzy and nauseous. These initial responses to nicotine have significant impacts on sustained smoking and subsequent development of nicotine dependence^15^,^16^,^17^,^18^. However, in a typical genetic study, the psychological effects of nicotine are rarely utilized and included in association testing. This omission is due, in part, to the fact that little is known of the relationship between these effects and genetic risks of nicotine dependence. In the literature, there are a few reports on the association between subjective measures and genetic variants in the nicotinic receptors, and some nominal associations are found in the CHRNB3/CHRNA6^19^ and CHRNB2^20^ genes. These studies use the scores of individual items or several items that contribute to a common factor, but these studies do not control for measurement errors and correlations between items, and do not have nicotine dose effect information.

Psychometric analysis with structural equation modeling provides a framework for constructing latent factor structures that can take into account measurement errors, item residual correlations and the degree of invariance across multiple waves of data simultaneously. In theory, it can identify and differentiate “true” treatment responses from confounding effects, attribute responses directly to experimental treatments (nicotine administration and dosage), and permit the test of genetic effects on these responses. Thus, integration of structural modeling with association testing may improve statistical power when well targeted hypotheses are postulated. To demonstrate the utility and advantage of this approach, we conducted a pilot study to evaluate the relationship between genetic risks of nicotine dependence and the subjective responses resulting from nicotine administration. We hypothesized that genetic risks of nicotine dependence contributed to subjective responses, and that by integrating psychometric modeling of psychological effects and dose effects we could improve statistical power to detect this genetic association. In a lab setting, we recruited 208 non-smokers, and measured their psychological responses via self-report by the Profile of Mood State (POMS)^21^, the Positive and Negative Affect Scale (PANAS)^22^, and the Direct Effects of Nicotine scale (DEN)^23^ after administration of each of a series of nicotine doses. We used the 3 SNPs (rs16969968, rs588765 and rs578776) that were shown to be associated independently with smoking quantity at the CHRNA5-CHRNA3-CHRNB4 locus^13^ as an example. In this article, we report the results of this pilot study.

## Results

Our primary goals were to evaluate how nicotine administration influenced subject's mood and affect as measured by the POMS, PANAS and DEN, and whether mood states and affect were influenced by genetic risks of nicotine dependence using the established markers from the CHRNA5-CHRNA3-CHRNB4 locus. Towards these goals, we needed first to examine the latent factor structures of the items of the instruments, and to evaluate whether the latent measurement factor structures remained equivalent (i.e. statistically invariant) after the administration of different nicotine doses using measurement invariance (MI) testing procedures. A failure in the MI testing would provide evidence that the latent factor(s) and the individual differences they represent were not constructed equivalently across the nicotine administrations, i.e., individual differences on the latent factors were not calibrated in the same way across administrations, presumably due to differential item responses attributable to the administration of different amounts of nicotine. Second, given the factor structures, we could test the association between the factors and genetic variants, and compare effects of the association across different dosage.

### Exploratory factor analyses of the POMS, PANAS and DEN

After removing poorly responded to items, we conducted exploratory factor analyses (EFA) for the POMS, PANAS and DEN item sets. In the analyses of the POMS, 24 items were included. Specifically, we conducted exploratory analyses for the 0 mg, 2 mg and 4 mg gum sessions separately, extracting 3-, 4-, 5- and 6-factor solutions respectively. A 5-factor solution fit the data adequately as judged by the root mean square of the residuals (RMSR), root mean square error of approximation (RMSEA), Tucker-Lewis Index (TLI) and χ^2^ test collectively (Table S1). For example, for the 0 mg nicotine administration, compared to the 4-factor solution, the 5-factor solution had better χ^2^ *P* value (0.0028 vs. 1.0E-6), RMSEA (0.045 vs. 0.057) and TLI (0.968 vs. 0.945), while the RMSR (0.03 vs. 0.03) was comparable. EFAs of the 2 and 4 mg of nicotine administration showed the same results. Further examination revealed that while the items consistently loaded onto the same factors (configural invariance), some loadings displayed differences across the 3 nicotine dosages (Table S2). The 5-factor solution included 3 positive and 2 negative factors. The first positive factor, labeled Friendly, consisted of 7 items (Friendly, Considerate, Efficient, Helpful, Cheerful, Good Natured and Trusting). The second positive factor, labeled Active, consisted of the items of Lively, Active, Energetic, Full of Pep and Vigorous. The third positive factor, labeled Clear Headed, was contributed by 3 items (Clear Headed, Unable to Concentrate (negative loading), and Alert). The negative factors were called On Edge (Tense, On Edge, Uneasy, Restless and Unable to Concentrate) and Fatigued (Worn out, Fatigued and Sluggish). It appeared that the loadings for the Friendly and Active factors changed significantly across the 3 nicotine gum doses while the Clear Headed, Uneasy and Fatigued factors remained stable (Table S2). In the literature, there was a report of a 5-factor structure of the POMS among smokers and non-smokers^24^. Generally, the POMS items typically are reported to have 6 distinct factors^21^,^25^. In our EFA using 6-factor solutions, Sympathetic and Care Free had substantial loadings (~0.5) on the 6^th^ factors, but other item's loadings on this sixth factor were below 0.2. For this reason, they were excluded from further analyses.

Similar analyses were done for the PANAS. The EFA results pointed to a 3-factor structure based on the RMRS, RMSEA, TLI and χ^2^ test (Table S3). The 3-factor model, after the administration of 0 mg of nicotine, had reasonable absolute fit (RMRS, 0.04) and comparative fit (TLI, 0.075). While the 4-factor model had better absolute and comparative fits comparing to the 3-factor model, no indicator had a reasonable factor loading (maximal λ = 0.42 for all indicators) on the fourth factor. The models for the 2 and 4 mg of nicotine showed the same pattern. Therefore, we decided on the 3-factor solution. The first positive affect factor, labeled Inspired, included the following items: Interested, Excited, Enthusiastic, Proud, Strong, Determined, Inspired and Active. The second positive factor, Attentive, had strong loadings for the items of Interested, Alert, Attentive and Active. Two items, Interested and Active, had substantial cross loading on both factors. The negative factor, Nervous, consisted of the items Distressed, Nervous, Jittery, Afraid and Irritable. The loadings of individual items were also consistent across the 3 nicotine doses (Table S4). Typically, the PANAS shows a 2-factor structure, but in different type of samples, latent structures of more than 2 factors have been reported^26^. We did not find any report of structural analysis of PANAS with smokers or with nicotine treatment.

The EFAs of the DEN suggested a 2-factor structure (Table S5). All 10 items had significant loadings (range from 0.23 ~ 0.78) on the first factor. Two items, Dizzy and Light Headed, had significantly higher loadings (0.82–0.94) on a second factor. The cross loadings between the two factors were substantial (Table S6). When forcing a single factor structure, the overall fit was worse than that of a two-factor solution (for example, for the models after administration of 0 mg of nicotine, 1-factor model: RMRS 0.06, TLI 0.873, and χ^2^ p value 1.9E-14; 2-factor model: RMRS 0.03, TLI 0.964, and χ^2^ p value 5.8E-3, Table S5). For this reason, we choose the 2-factor model in the subsequent analyses. The 2 factors were Confused and Dizzy. The Confused factor had 7 items (Nauseous, Nervous, Sweaty, Headache, Salivation, Heart Pound, and Confused), and the Dizzy factor had 3 items (Dizzy, Lite Headed and Weak).

### Invariance tests for the POMS, PANAS and DEN models

**Figure 1:**
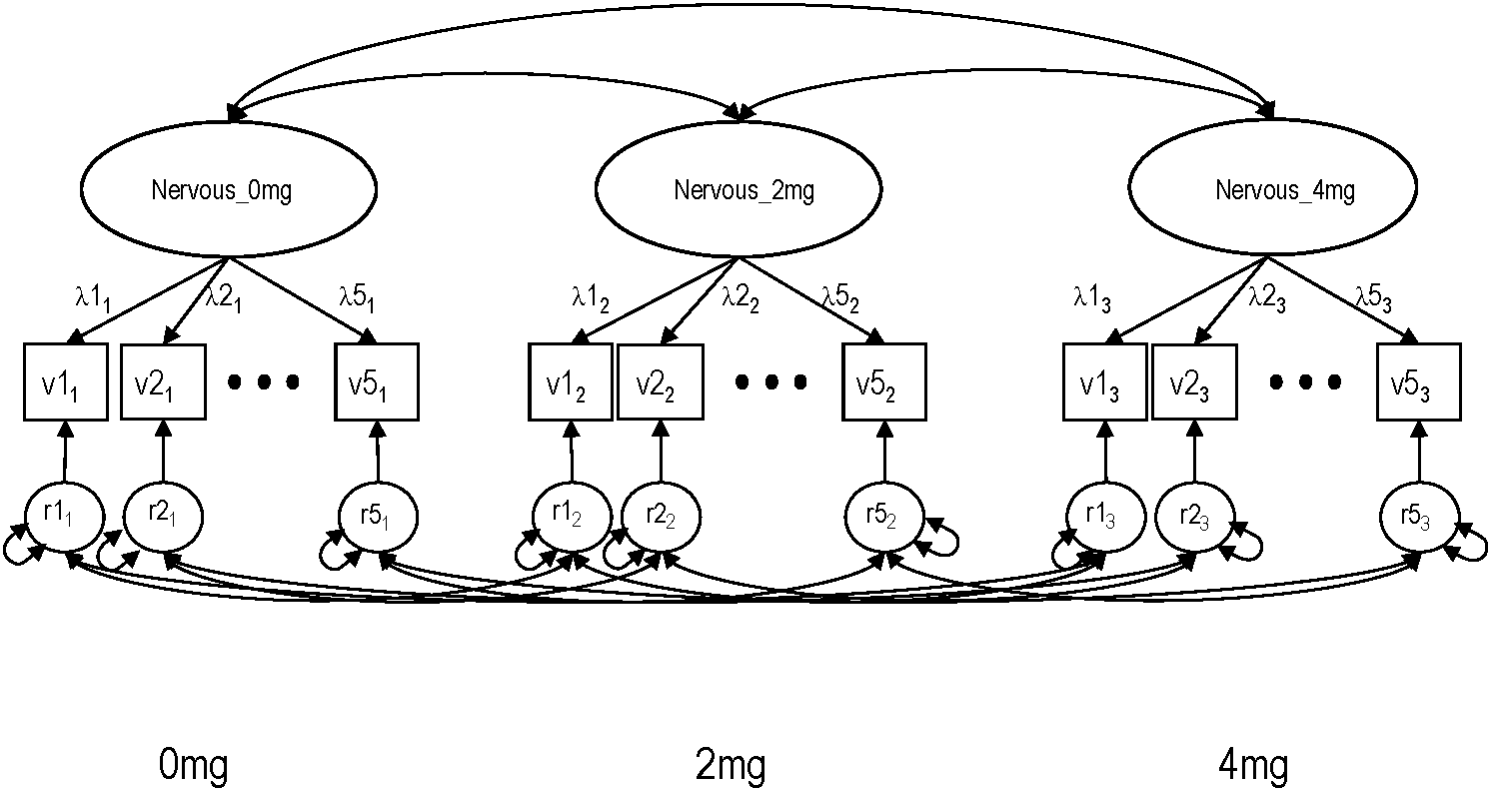
Configural latent variable measurement model for testing invariance across nicotine doses. The figure is shown using the PANAS Nervous factor.

Based on the results of EFA, confirmatory measurement models were constructed for each of the 3 nicotine administrations for the POMS, PANAS and DEN instruments. Since our sample size was modest, to avoid over parameterization, models for the positive and negative factors were modeled separately. In these common factor models for each administration, correlations among the same item's residuals were allowed across the 3 nicotine doses since assessments were obtained within relatively short time intervals leaving open the possibility of memory carry over. Inter-factor correlations across the doses and between different affect factors (since POMS had 3 positive factors and 2 negative factors, and PANAS had 2 positive factors) were also included. A path diagram of the general measurement model is shown in Figure 1 using the PANAS Nervous factor as an example.

Following general guidelines for MI testing, configural invariance was first examined using the confirmatory factor analyses (CFA). Based on the factor models derived from the EFAs, metric (factor loadings) and scalar (thresholds/intercepts) invariance were imposed across the 3 nicotine doses sequentially, and compared to the saturated baseline model using RMSEA, TLI, BIC and χ^2^ test. The results of the MI tests for the POMS were summarized in Tables 1. The overall model fits of the configural models for POMS were reasonable (three positive factors, CFI 0.994, TLI 0.993, RMSEA from 0.078; two negative factors, CFI 0.999, TLI 0.999, RMSEA 0.024). When the factor loadings were forced to be invariant across the 3 nicotine doses, the change of χ^2^ was insignificant for the positive factors (Δ χ^2^ 10.5, Δ DF 29, p = 0.9993), and the change of χ^2^ was nominally significant (Δ χ^2^ 32.2, Δ DF 18, p = 0.0208) for the negative factors. Further constraining the thresholds led to highly significant misfits for both positive and negative factors (positive factors, Δ χ^2^ 397.1, Δ DF 116, p < 2.2E-16; negative factors, Δ χ^2^ 444.5, Δ DF 52, p < 2.2E-16). The overall fit indices were reasonable (CFI 0.988–0.999, TLI 0.988–0.999, RMSEA 0.024–0.078).

Similar results for the PANAS and DEN were observed (Tables 2 and 3). For the PANAS items, the positive factors model also failed the metric invariance test (Δ χ^2^ 57.9, Δ DF 24, p = 1.24E-4). Overall, the failure of MI tests in all instruments at the metric and/or scalar levels indicated that nicotine administration resulted in differential item functioning for the mood and affect factors measured by the POMS, PANAS and DEN. In other words, we found evidence that volunteers responded differentially to some of the items in these instruments after administration of different doses of nicotine, resulting in the change of factor structure of these instruments. These results provided a solid foundation for testing the association of these nicotine specific effects with genetic variants associated with nicotine dependence.

### The association of genetic risks of nicotine dependence and latent factors

**Figure 2:**
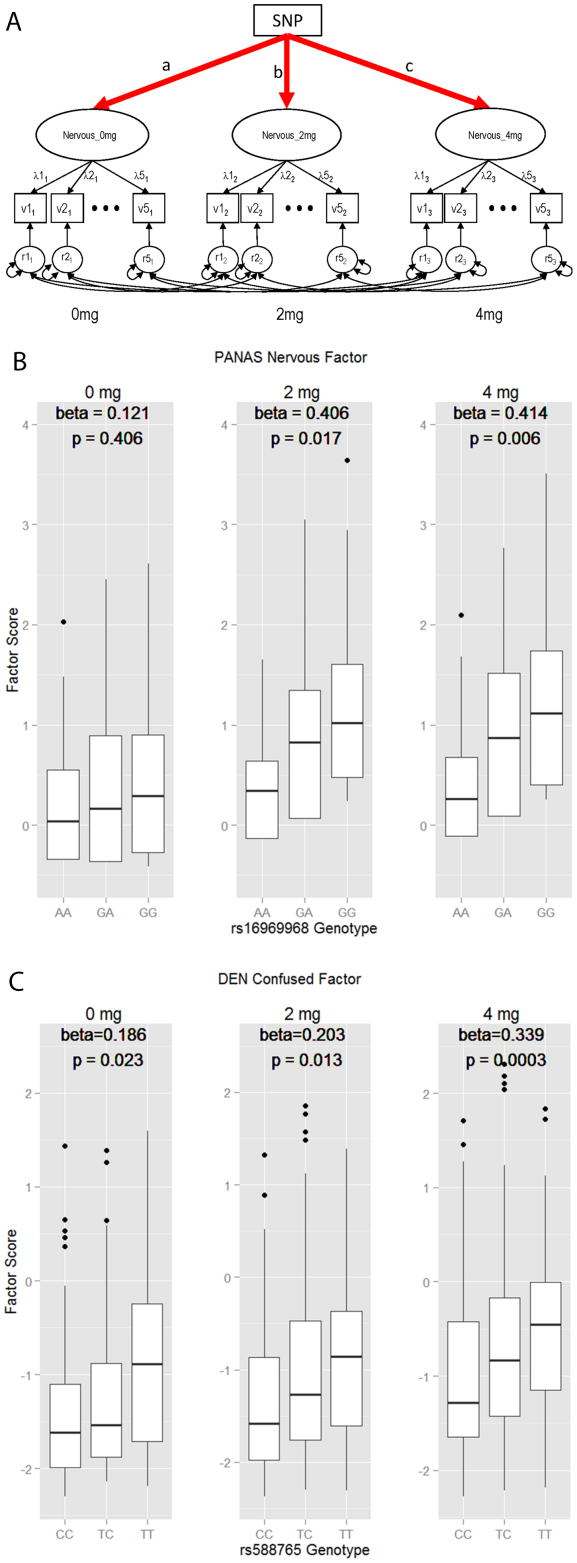
Measurement model based association testing between SNP genotypes and the means of latent factors. (A). The invariant base model used in the testing. (B). The association between rs16969968 genotypes and the scores of the PANAS Nervous factor. (C). The association between rs588765 genotypes and the scores of the DEN Confused factor.

Having shown that nicotine administration was related to differential influences on subjects' mood and affect, we proceeded to test if known genetic risks to developing nicotine dependence could predict variation in these psychological measures. We selected 3 SNPs from the CHRNA5-CHRNA3-CHRNB4 locus to test the association between the latent factors identified in our item level analyses and the genotypes of these SNPs. In these models, the factor loadings and thresholds/intercepts of all items were held invariant across the 3 nicotine doses (i.e., as in scalar invariance test, Figure 2A), forcing the effects of nicotine administration to be represented at the latent factors. Under these conditions, we regressed the latent factors onto the SNP genotypes, testing whether SNP genotypes could linearly predict variation in the latent mood factors. The results were summarized in Tables 4–6 for POMS, PANAS and DEN respectively. Regression results suggested that the positive factors and negative factors had different patterns. For the POMS positive factors, the Active factor demonstrated nominally significant association with rs16969968, the most significant finding at the CHRNA5-CHRNA3-CHRNB4 locus. For the negative factors, most of them showed an association with genotypes, although the strength of association was modest. SNP rs588765 was associated with 4 of the 5 negative factors identified in this study. Rs16969968 had association with 3 of the 5 factors. These results indicated that genetic markers associated with nicotine dependence predicted variations in negative mood and affect in current non-smokers, the cohort used in this study. To visualize the results, we computed the Nervous factor scores for the PANAS dataset and plotted them against the genotypes of rs16969968 (Figure 2B). A similar plot was made for the DEN Confused factor and rs588765 (Figure 2C). Of note, subjects carrying the risk alleles (the A allele of rs16969968 and the C allele of rs588765) had lower negative factor scores, indicating that these subjects tended to be less negative on nicotine ingestion as compared to subjects not carrying the risk alleles.

In these analyses, we tested the association of 3 SNPs with the latent factors identified in our measurement models across 3 nicotine doses. The factors from the POMS, PANAS and DEN measured different aspects of mood and affect, but they were not independent. For example, the correlation between the factors varied from 0.3 to 0.9 for the DEN. The effects of nicotine on these factors were most likely accumulative, i.e. the effects observed at 4 mg were the sum of 2 and 4 mg of nicotine, because it took 30–45 min to reach maximal plasma nicotine concentration after gum administration^27^,^28^. How to correct for multiple testing was an important issue in evaluating the significance of these tests. If we combined all positive and negative factors in each instrument, and considered the 3 SNPs independent, we needed to correct for 15 tests (2 positive factors and 3 negative factors, for 3 SNPs). Under these conditions, rs588765 was associated with DEN factors (Confused at 4 mg, p = 0.0003 and Dizzy at 0 mg, p = 0.001). rs588765 and rs16969968 showed a trend for the PANAS Nervous at 4 mg (p = 0.006 and 0.007 respectively). None of the positive factors survived this correction.

To evaluate how nicotine dose influenced the mood and affective states of volunteers, we conducted *post hoc* analyses for the DEN factors that showed significant association with genotypes. In these analyses, we compared the fit indices between two models: the SNP model, with scalar invariance and SNP as exogenous predictor (Figure 2A), and a constrained SNP model, which added constraints on the regression coefficients of SNP prediction by setting paths a, b, and c in Figure 2A to be invariant. The results were summarized in Table 7. When the regression coefficients were forced to be equal across the 3 nicotine doses, it caused significant misfit of the constrained model for SNP rs588765, the marker showing significant association with the factors in this study. These results implied that the regression coefficients (effect sizes) for rs588765 on DEN factors were statistically different across the nicotine doses. Since the coefficients increased from 0 mg to 4 mg (Table 6), it could be interpreted that the change of effect sizes was proportional to the amount of nicotine administered.

## Discussion

In this study, current non-smokers were recruited to examine the effects of nicotine administration on their responses to the POMS, PANAS and DEN instruments. Our objectives were to evaluate whether previously identified genetic risks for nicotine dependence (i.e., SNPs at the CHRNA5 locus) predicted variation of self-reported subjective states using psychometric analyses with structural equation modeling. We performed exploratory and confirmatory factor analyses to determine the factor structures of these instruments. Based on these structures, we carried out MI testing to evaluate if the administration of increasing levels of nicotine had an impact on the structures. We further tested whether known genetic variants were associated with these affective factors. The main findings of our study were two-fold: 1) Using increasing nicotine doses and repeated measures with standard instruments, we found that nicotine administration had significant differential impacts on the mood measurement structures for both positive and negative affect in current non-smokers, implying that nicotine dose had direct and measurable effects on items assessing mood and affect; and 2) Variation in mood and affect states under forced MI models were predicted by previously identified genetic risk factors of nicotine dependence, suggesting that the effects of nicotine on mood and affect were linked to genetic risks of nicotine dependence. Furthermore, *post hoc* analyses of rs588765 suggested that the effect might be proportional to the amount of nicotine administered.

Our findings have important implications. Traditionally, genetic studies of nicotine dependence use smokers (current smokers and/or ex-smokers), and phenotype ascertainments focus on behavioral outcomes (e.g., number and/or duration of cigarette use). The effects of nicotine as a drug on mood and affect are rarely incorporated. Our study is the first, to our knowledge, to provide direct evidence that psychological effects of nicotine can be used for genetic studies of nicotine dependence in current non-smokers. When we designed the study, we decided to use current non-smokers to avoid the complication of neuroadaptive changes associated with cigarette smoking^29^. We reasoned that subjects with minimal exposure would be better to measure the psychological effects of nicotine. Can we use these same psychological effects from smokers for genetic studies of nicotine dependence? A few reports in the literature suggest yes^19^,^30^,^31^. However, we do not know whether the effects on smokers are more or less informative compared to that of current non-smokers. Therefore, a similar study in smokers would be of interest.

Interestingly, we were able to detect consistent association signals in a sample of 208 subjects that might otherwise require a sample size several times larger in a typical genetic association study. This indicates that the integration of the drug effects of nicotine with psychometric structural modeling can be a powerful approach in genetic studies of nicotine dependence. In genetic studies, several factors influence the power. In addition to sample size, the reliability and accuracy of phenotype measurements are one of the most important, but often overlooked, factors to improve power for a given trait. In a typical study of psychiatric trait, phenotypic data are normally based on self-reports and the accuracy of these self-reports are far from satisfactory. For this reason, researchers have used psychometric models to obtain factor scores to serve as the phenotype^32^,^33^. In the studies of other complex diseases such as cardiovascular diseases, researchers use biologically relevant surrogates (i.e. endophenotypes)^34^ to improve power. Specific to nicotine dependence study, cotinine, a major metabolite of nicotine, has been used as a surrogate, and the study has demonstrated tremendous improvement in statistical power^11^. In our study, we used psychometric analyses of multiple waves of data to control for measurement errors and confounding effects, achieving experiment-wide significance for rs588765. In a study with 789 nicotine dependence cases and 811 controls^19^, subjective responses similar to the DEN were used as phenotypes. Although the study found associations between the CHRNB3 gene and the “dizziness” phenotype, no associations were found for any markers typed in the CHRNA5-CHRNA3-CHRNB4 locus, including rs16969968 and rs578776, despite the much larger sample size. In contrast, with only 208 subjects, we found nominal association between rs16969968 and the Dizzy factor. The gains in power in our study, we believe, is partly due to the integration of psychometric modeling of item level data for multiple waves of measurement which effectively accounts for measurement errors and confounding effects. Another advantage of integration of psychometric modeling with experimentally generated data is that it provides a much stronger basis for interpreting the results. What the modeling effects might be attributed to is more clearly defined and circumscribed, i.e. the controlled administration of nicotine in this study.

We also notice that while there are quantifiable differences for both positive and negative effects across increasing doses of nicotine, genetic associations are observed largely on the negative factors. This may be due to the fact that our subjects are non-smokers, or because negative affect factors have larger effect sizes. In the literature, there is a suggestion that non-smokers (including both never smokers and ex-smokers) react to smoking more negatively than smokers do^24^. In this study, we did find that ever smokers had higher negative factor scores than the never smokers, although the differences were not statistically significant (data not shown). This greater negative affect among the ever smokers toward nicotine administration may play a role in their persistent abstinence.

This study has some limitations. Given the relatively small sample size, while modest genetic effects are detected, most estimates do not survive corrections for multiple comparisons. However, the observed effects of SNPs are consistent across all doses of nicotine and for all three subjective instruments; therefore, our modest associations are more likely due to a small sample size rather than Type II error. Nonetheless, further studies are needed to replicate and verify our findings. The small sample size also prevents us from expanding to more complex models to estimate the trajectory of the effects of nicotine intake, and to test if the amount of change is proportional to nicotine doses and if it is influenced by genetic risks of nicotine dependence. These are of great interest for the research community, and we hope to extend the study to address these questions.

In summary, in this study, we provide the first evidence linking established genetic risk factors for nicotine dependence to mood states and affect of non-smokers under controlled laboratory conditions. These results demonstrate that the psychological effects of nicotine as a drug can, and should be utilized in genetic studies of smoking addiction and nicotine dependence. Study designs incorporating these effects may be more powerful than typical studies using only smoking behaviors as phenotypes.

## Methods

### Subjects

Non-smokers of European ancestry between the ages of 18 and 50 years old were recruited to participate in the study. We defined non-smokers as those individuals who reported not smoking in the last 3 months and also smoking less than 100 cigarettes during their lifetime. The cutoff of 100 cigarettes smoked in one's lifetime is commonly used to differentiate between “smokers” and “nonsmokers”^35^,^36^. Current smoking status was verified by breath carbon monoxide (CO) level (7 ppm or lower, BreathCO; Vitalograph, Lenexa, KS), and by urine cotinine concentration (2 ng/mL or less). Individuals were excluded if they reported a history of chronic health problems or psychiatric conditions, were pregnant or breastfeeding, or had other major medical conditions. The total number of subjects included for the study was 208, with an average age of 26 years (standard error, se 0.49) and average years of education of 15.5 (se 0.14). One hundred and eight were females (52.7%), and 102 (49.2%) reported having smoked some cigarettes (i.e. these subjects smoked some cigarettes in their lifetime, but they did not smoke in the last 3 months and the total number of cigarettes smoked was less than 100 cigarettes). The study was reviewed and approved by the IRB of Virginia Commonwealth University. All subjects provided informed consent to participate in this study. The experiments were conducted in accordance with procedures approved by the IRB.

### Lab procedures

After providing informed consent, all volunteers participated in a single laboratory session that included the administration of three doses of nicotine gum in ascending dose order (0, 2, and 4 mg) and measurement of self-reported mood and affect responses. Upon arrival to the laboratory, subjects were connected to a physiological monitor (Model 507E, Criticare Systems) for continuous recording of heart rate and blood pressure. Baseline self-report ratings using the POMS, PANAS and DEN were also obtained. Participants were then administered the first piece of nicotine gum (0 mg) and instructed to follow a computer-guided pacer to chew the gum for 15 minutes^37^,^38^. Immediately after the gum was discarded, participants again were asked to respond to the subjective questionnaire items. This same procedure (15 minute standardized gum chewing period followed by subjective questionnaires) was repeated for the 2 and 4 mg doses of nicotine gum, with 45 minutes in between each administration. Blood samples were drawn prior to the first gum administration for genotype analyses.

### Outcome measures

We used the POMS, PANAS and DEN questionnaires to evaluate the effects of nicotine administration on mood and affect. In this study, we used POMS, a 65-item, single-word questions to assess each subject's current mood states^21^. For each question, a 5-level Likert type response format was used to record the level of his/her affect at the time. The PANAS is a 20-item single-word questionnaire designed to obtain positive and negative affect responses using a 5-level response format^22^. The DEN is a 10-item single-word instrument tapping more physiologically based aspects of nicotine effects (e.g., “dizzy”)^23^. Participants indicated their response to each item on a computerized scale ranging from 0 to 100 (whole numbers) using the computer pointer.

### DNA extraction and genotyping

DNAs were extracted from blood samples using a kit (Qiagen, QiaAmp DNA Blood Midi kit, Valencia, CA 91355) and manufacture provided protocol. After the extraction, DNA samples were quantified and stored at −80°C. Genotypes for the subjects for rs16969968, rs588765 and rs578776 were obtained using the TaqMan method^39^. The genotype and allele frequencies of the 3 markers were in Hardy-Weinberg equilibrium.

### Data analyses

The POMS and PANAS items, and SNP genotype data were modeled as ordered categorical (ordinal) variables. For the POMS and PANAS, items that showed limited variability with more than 90% of subjects using only one of the response categories were dropped from further analyses. Twenty four items of the POMS and 15 items of the PANAS were retained and included in this analysis. For the 10 DEN items, a log 10 transformation was performed in an attempt to normalize their distributions. All 10 items were included in the analyses. First, exploratory item factor analyses were conducted for the POMS, PANAS and DEN item sets using the R packages PSYCH and POLYCOR with oblimin rotation to determine the appropriate dimensionality of the latent structures of each set of items. Second, measurement invariance (MI) testing of the POMS, PANAS and DEN structures were performed with the R packages LAVAAN^40^ and SemTools. MI testing was carried out to evaluate if the affect factor measurement structures remained invariant across the different nicotine gum administrations. Third, the associations of SNPs with the psychological latent factors were tested within models where the loadings and thresholds/intercepts of the indicators of each latent factor were constrained to be equal across the 3 nicotine administrations. This approach tests whether changes in factor scores are confounded with any differential item functioning that may be present. Therefore, the tests of association of the SNPs with the psychological factors provide estimates of unambiguous effects indicating whether the genetic risk factors (i.e., SNPs) predict variation and changes in psychological status in the context of the effects of different doses of nicotine administration.

## Supplementary Material

Supplementary Information Supplementary information

## Figures and Tables

**Table 1:** Summary of measurement invariance test of the POMS models

Model	DF	χ^2^	Δ χ^2^	Δ DF	Δ χ^2^ P	CFI	TLI	RMSEA
POMS Positive Factors (“Friendly”, “Active” and “Clear Headed”)								
Configural model	879	1990.5				0.994	0.993	0.078
Metric Invariance	908	2043.9	10.5	29	0.9993	0.994	0.994	0.078
Scalar Invariance	1024	2229.3	397.1	116	**<2.2E-16**	0.994	0.994	0.076
POMS Negative Factors (“On Edge” and “Fatigued”)								
Configural model	218	242.9				0.999	0.999	0.024
Metric Invariance	236	297.8	32.2	18	**0.0208**	0.997	0.997	0.036
Scalar Invariance	288	577.6	444.5	52	**<2.2E-16**	0.988	0.988	0.070

CFI: comparative fit index.

**Table 2:** Summary of measurement invariance test of the PANAS models

Model	DF	χ^2^	Δ χ^2^	Δ DF	Δ χ^2^ P	CFI	TLI	RMSEA
PANAS Positive Factors (Inspired and Attentive)								
Configural model	364	707.9				0.997	0.997	0.068
Metric Invariance	388	832.5	57.9	24	**1.24E-04**	0.996	0.996	0.075
Scalar Invariance	468	904.7	175.6	80	**3.98E-09**	0.997	0.997	0.067
PANAS Negative Factor (Nervous)								
Configural model	77	121.2				0.991	0.988	0.053
Metric Invariance	85	142.5	10.3	8	0.2458	0.989	0.986	0.057
Scalar Invariance	107	267.0	197.6	22	**<2.2E-16**	0.969	0.969	0.085

**Table 3:** Summary of measurement invariance test of the DEN models

Model	DF	AIC	BIC	χ^2^	Δ χ^2^	Δ DF	Δ χ^2^ P	CFI	TLI	RMSEA
Configural model	409	7779.7	8267.0	1000.9				0.873	0.846	0.083
Metric Invariance	428	7777.6	8201.4	1036.7	26.8	13.5	**0.0168**	0.869	0.849	0.083
Scalar Invariance	444	7845.2	8215.7	1136.4	99.1	16.7	**9.97E-14**	0.851	0.834	0.087

AIC: Akaiki information criterion.

**Table 4:** Association of POMS factors with SNPs*

	Placebo (0 mg)				Nicotine Gum (2 mg)				Nicotine Gum (4 mg)			
SNP	Beta	Se	Z	P	Beta	Se	Z	P	Beta	Se	Z	P
Friendly												
rs16969968	0.065	0.110	0.593	0.5530	0.071	0.108	0.655	0.5120	0.065	0.114	0.569	0.5690
rs578776	0.126	0.110	1.146	0.2520	*0.181*	*0.109*	*1.671*	*0.0950*	0.155	0.111	1.397	0.1620
rs588765	−0.023	0.102	−0.22	0.8230	−0.038	0.100	−0.375	0.7080	−0.039	0.103	−0.380	0.7040
Active												
rs16969968	*0.189*	*0.110*	*1.720*	*0.0850*	**0.261**	**0.110**	**2.378**	**0.0170**	**0.228**	**0.115**	**1.977**	**0.0480**
rs578776	0.095	0.110	0.866	0.3870	*0.205*	*0.110*	*1.856*	*0.0630*	0.155	0.118	1.320	0.1870
rs588765	0.011	0.102	0.110	0.9130	0.031	0.100	0.308	0.7580	0.049	0.105	0.466	0.6420
Clear Headed												
rs16969968	−0.106	0.125	−0.85	0.3970	−0.061	0.130	−0.472	0.6370	0.002	0.126	0.019	0.9850
rs578776	0.082	0.130	0.636	0.5250	0.083	0.115	0.721	0.4710	0.115	0.127	0.907	0.3640
rs588765	−0.111	0.116	−0.957	0.3390	−0.041	0.116	−0.348	0.7270	0.004	0.121	0.031	0.9750
Uneasy												
rs16969968	0.164	0.134	1.220	0.2220	0.166	0.123	1.349	0.1770	0.132	0.120	1.099	0.2720
rs578776	−0.188	0.138	−1.366	0.1720	−0.017	0.126	−0.134	0.8930	*−0.218*	*0.119*	*−1.830*	*0.0670*
rs588765	*0.236*	*0.129*	*1.822*	*0.0690*	0.109	0.126	0.861	0.3890	**0.243**	**0.121**	**2.013**	**0.0440**
Fatigued												
rs16969968	0.132	0.128	1.032	0.3020	−0.013	0.124	−0.107	0.9150	−0.045	0.123	−0.368	0.7130
rs578776	0.090	0.121	0.750	0.4530	−0.065	0.126	−0.517	0.6050	−0.109	0.126	−0.865	0.3870
rs588765	0.056	0.114	0.491	0.6230	0.048	0.120	0.398	0.6900	0.037	0.115	0.321	0.7480

*P values ≤ 0.05 were highlighted by bold fonts, and p values ≤ 0.1 were highlighted by italic fonts and underscored.

**Table 5:** Association of PANAS factors with SNPs

	Placebo (0 mg)				Nicotine Gum (2 mg)				Nicotine Gum (4 mg)			
SNP	Beta	Se	Z	P	Beta	Se	Z	P	Beta	Se	Z	P
Inspired												
rs16969968	0.074	0.110	0.673	0.5010	0.154	0.111	1.389	0.1650	0.148	0.115	1.286	0.1990
rs578776	0.104	0.113	0.916	0.3600	0.151	0.112	1.340	0.1800	0.141	0.111	1.268	0.2050
rs588765	−0.044	0.102	−0.44	0.6630	−0.005	0.104	−0.04	0.9650	0.046	0.106	0.430	0.6670
Attentive												
rs16969968	0.053	0.123	0.433	0.6650	0.108	0.115	0.939	0.3480	0.082	0.122	0.677	0.4980
rs578776	0.095	0.113	0.843	0.3990	0.130	0.106	1.218	0.2230	0.139	0.113	1.230	0.2190
rs588765	0.002	0.104	0.023	0.9820	−0.001	0.104	−0.013	0.9900	−0.017	0.109	−0.159	0.8740
Nervous												
rs16969968	0.121	0.146	0.830	0.4060	**0.406**	**0.169**	**2.394**	**0.0170**	**0.414**	**0.149**	**2.768**	**0.0060**
rs578776	−0.150	0.155	−0.966	0.3340	0.082	0.14	0.587	0.5570	−0.062	0.135	−0.460	0.6460
rs588765	*0.240*	*0.136*	*1.763*	*0.0780*	0.218	0.148	1.471	0.1410	**0.367**	**0.136**	**2.690**	**0.0070**

**Table 6:** Association of DEN factors with SNPs

	Placebo (0 mg)				Nicotine Gum (2 mg)				Nicotine Gum (4 mg)			
SNP	Beta	Se	Z	P	Beta	Se	Z	P	Beta	Se	Z	P
Confused												
rs16969968	−0.119	0.078	−1.528	0.1270	*−0.158*	*0.082*	*−1.941*	*0.0520*	**−0.213**	**0.101**	**−2.103**	**0.0350**
rs578776	−0.123	0.076	−1.608	0.1080	−0.134	0.092	−1.446	0.1480	−0.165	0.103	−1.591	0.1120
rs588765	**0.186**	**0.082**	**2.266**	**0.0230**	**0.203**	**0.082**	**2.472**	**0.0130**	**0.339**	**0.094**	**3.598**	**0.0003**
Dizzy												
rs16969968	**−0.198**	**0.079**	**−2.498**	**0.0120**	**−0.267**	**0.092**	**−2.884**	**0.0040**	−0.118	0.105	−1.121	0.2620
rs578776	−0.096	0.080	−1.204	0.2280	0.146	0.090	1.616	0.1060	−0.019	0.089	−0.212	0.8320
rs588765	**0.247**	**0.075**	**3.276**	**0.0010**	0.152	0.087	1.744	0.0810	**0.183**	**0.090**	**2.035**	**0.0420**

**Table 7:** *Post hoc* analyses of nicotine dose on the DEN factors

SNP	Model*	Df	χ^2^	Δ χ^2^	Δ DF	Δ χ^2^ P	CFI	TLI	RMSEA
rs16969968	SNP	476	1275.7				0.828	0.811	0.090
	Constrained SNP	480	1280.3	5.0	3.8	0.2650	0.828	0.812	0.090
rs578776	SNP	476	1291.3				0.826	0.808	0.091
	Constrained SNP	480	1299.4	7.2	3.7	0.1056	0.825	0.808	0.091
rs588765	SNP	476	1279.5				0.828	0.810	0.090
	Constrained SNP	480	1288.8	9.6	3.7	**0.0396**	0.827	0.810	0.090

*see text for full description of the models.
