# Supplementary material for: Genetic Risks to Nicotine Dependence Predict Negative Mood and Affect in Current Non-Smokers
Source: Sci Rep. 2015 Mar 31;5:9521. doi: 10.1038/srep09521 (PMC4894432; doi:10.1038/srep09521)
Supplement: Supplementary Information [file srep09521-s1.pdf]

Genetic Risks to Nicotine Dependence Predict Negative Mood and Affect in Current Non-Smokers

Xiangning Chen,[1,2] Steven H Aggen,[1] Jingchun Chen[1], Lingxi Li,[1] Kenneth S Kendler[1,2], Melissa Blank,[3] and Thomas Eissenberg[4]

1: Virginia Institute for Psychiatric and Behavioral Genetics, Virginia Commonwealth University;

2: Department of Human and Molecular Genetics, Virginia Commonwealth University;

3: Department of Psychology, West Virginia University

4: Department of Psychology and Center for the Study of Tobacco Products, Virginia Commonwealth University

Supplementary Information

Table S1. Exploratory factor analyses of the POMS

	model	# factors	RMSR	RMSEA	TLI	χ^2 P
0 mg	POMS_1	3	0.04	0.078	0.891	6.60E-19
	POMS_2	4	0.03	0.057	0.945	1.00E-06
	**POMS_3**	**5**	**0.03**	**0.045**	**0.968**	**0.0028**
	POMS_4	6	0.02	0.039	0.977	0.0280
2 mg	POMS_1	3	0.04	0.087	0.865	6.80E-27
	POMS_2	4	0.04	0.071	0.913	5.50E-13
	**POMS_3**	**5**	**0.03**	**0.058**	**0.943**	**1.50E-06**
	POMS_4	6	0.02	0.042	0.973	0.012
4 mg	POMS_1	3	0.04	0.096	0.855	1.90E-35
	POMS_2	4	0.03	0.068	0.931	3.40E-11
	**POMS_3**	**5**	**0.03**	**0.059**	**0.948**	**8.20E-07**
	POMS_4	6	0.02	0.048	0.969	0.0018

RMSR: root mean square of the residuals
RMSEA: root mean square error of approximation
TLI: Tucker-Lewis index
The models highlighted by bold fonts were selected for subsequent confirmatory analyses and MI testing.

Table S2. Factor loadings of the POMS

	0 mg gu m							2 mg gum							4 mg gum						
**Items**	Friendly	OnEdge	Fatigued	Active	ClearHeaded	h2	u2	Friendly	OnEdge	Fatigued	Active	ClearHeaded	h2	u2	Friendly	OnEdge	Fatigued	Active	ClearHeaded	h2	u2
Friendly	**0.77**	-0.18	-0.05	0.03	0.15	0.66	0.34	**0.77**	-0.15	-0.05	0.18	0.23	0.70	0.30	**0.83**	-0.16	-0.08	0.21	0.12	0.79	0.21
Tense	-0.10	**0.67**	0.12	0.03	0.01	0.48	0.52	-0.08	**0.67**	0.18	-0.02	0.01	0.49	0.51	-0.14	0.75	0.12	-0.04	0.07	0.6	0.4
WornOut	-0.08	0.11	**0.91**	0.03	0.03	0.84	0.16	-0.09	0.21	**0.86**	-0.01	0.10	0.81	0.19	-0.10	0.26	**0.85**	-0.15	0.04	0.82	0.18
ClearHeaded	0.55	-0.21	-0.18	0.12	**0.50**	0.65	0.35	0.45	-0.24	-0.05	0.17	**0.49**	0.53	0.47	0.33	-0.29	-0.11	0.30	**0.51**	0.56	0.44
Lively	**0.68**	-0.07	-0.20	**0.45**	0.08	0.72	0.28	**0.55**	-0.03	-0.15	**0.63**	0.10	0.74	0.26	**0.47**	-0.12	-0.16	**0.68**	0.08	0.73	0.27
Considerate	**0.81**	-0.03	-0.05	-0.05	0.09	0.66	0.34	**0.77**	-0.04	0.01	0.10	0.05	0.61	0.39	**0.82**	-0.03	-0.13	0.18	0.15	0.75	0.25
Active	**0.64**	0.02	-0.09	**0.51**	0.13	0.69	0.31	**0.50**	0.00	-0.06	**0.66**	0.13	0.71	0.29	**0.40**	0.01	-0.11	**0.73**	0.15	0.74	0.26
OnEdge	-0.07	**0.78**	0.06	-0.02	0.10	0.63	0.37	-0.05	**0.85**	0.07	-0.01	0.00	0.73	0.27	-0.08	**0.86**	0.04	-0.01	0.00	0.75	0.25
Energetic	**0.72**	-0.10	-0.22	**0.49**	0.13	0.84	0.16	**0.61**	0.01	-0.26	**0.59**	0.14	0.81	0.19	**0.40**	0.03	-0.31	**0.79**	0.07	0.88	0.12
Sympathetic	**0.64**	0.10	-0.03	0.12	-0.07	0.44	0.56	**0.58**	0.08	-0.02	0.21	-0.02	0.39	0.61	**0.60**	0.00	-0.01	0.26	0.02	0.43	0.57
Uneasy	-0.02	**0.79**	0.12	-0.07	-0.01	0.65	0.35	-0.18	**0.82**	0.13	0.02	-0.07	0.72	0.28	-0.22	**0.69**	0.23	-0.04	-0.08	0.59	0.41
Restless	-0.04	**0.68**	0.05	0.05	-0.09	0.48	0.52	0.05	**0.46**	0.10	-0.03	-0.16	0.25	0.75	0.00	**0.70**	0.06	-0.01	-0.12	0.51	0.49
Unab2conc	0.02	**0.55**	0.27	-0.05	**-0.27**	0.45	0.55	-0.08	**0.42**	0.31	-0.04	**-0.48**	0.50	0.50	-0.07	**0.55**	0.38	-0.07	**-0.43**	0.64	0.36
Fatigued	-0.12	0.16	**0.84**	-0.08	-0.09	0.75	0.25	-0.15	0.23	**0.82**	-0.10	-0.16	0.78	0.22	-0.11	0.19	**0.87**	-0.18	-0.02	0.83	0.17
Helpful	**0.74**	-0.01	-0.11	0.04	0.14	0.59	0.41	**0.70**	0.09	-0.09	0.24	0.16	0.58	0.42	**0.70**	-0.10	-0.05	0.23	0.13	0.57	0.43
Cheerful	**0.79**	-0.17	-0.07	0.06	0.05	0.66	0.34	**0.71**	-0.15	-0.15	0.19	0.11	0.60	0.40	**0.67**	-0.20	-0.20	0.35	0.07	0.66	0.34
GoodNat	**0.84**	-0.11	0.04	-0.11	-0.08	0.74	0.26	**0.85**	-0.17	-0.03	-0.02	0.03	0.76	0.24	**0.81**	-0.19	-0.08	0.20	0.11	0.75	0.25
Sluggish	-0.08	0.32	**0.64**	-0.13	-0.08	0.54	0.46	-0.09	0.18	**0.77**	-0.19	-0.18	0.70	0.30	-0.16	0.08	**0.70**	-0.27	-0.26	0.66	0.34
Alert	**0.67**	-0.05	-0.12	0.12	**0.49**	0.72	0.28	**0.58**	-0.12	-0.23	**0.29**	**0.50**	0.74	0.26	**0.50**	-0.01	-0.20	**0.47**	**0.38**	0.65	0.35
Efficient	**0.64**	-0.09	-0.06	0.14	0.25	0.50	0.50	**0.71**	-0.07	-0.09	0.25	0.23	0.63	0.37	**0.69**	-0.10	-0.14	0.38	0.15	0.68	0.32
Trusting	**0.73**	-0.01	0.07	-0.11	0.04	0.54	0.46	**0.71**	-0.06	-0.06	-0.01	0.05	0.52	0.48	**0.66**	-0.06	-0.06	0.13	-0.05	0.47	0.53
Fullopep	**0.72**	-0.01	-0.18	**0.41**	0.09	0.73	0.27	**0.67**	-0.06	-0.27	**0.49**	0.02	0.77	0.23	**0.51**	-0.07	-0.24	**0.68**	0.05	0.78	0.22
Carefree	**0.62**	-0.09	-0.09	0.20	-0.08	0.44	0.56	**0.51**	-0.13	-0.09	0.25	-0.11	0.36	0.64	**0.49**	-0.19	-0.09	0.32	-0.07	0.4	0.6
Vigorous	**0.61**	0.07	-0.06	**0.22**	0.05	0.44	0.56	**0.55**	0.01	-0.16	**0.33**	0.08	0.45	0.55	**0.48**	-0.01	-0.22	**0.50**	0.04	0.53	0.47
SS Loadings	7.96	2.76	2.26	1.09	0.79			6.83	2.54	2.46	2.00	1.03			6.01	2.95	2.57	3.40	0.83		
Proportion Variance	0.33	0.11	0.09	0.05	0.03			0.28	0.11	0.10	0.08	0.04			0.25	0.12	0.11	0.14	0.03		
Cumulative Variance	0.33	0.45	0.54	0.59	0.62			0.28	0.39	0.49	0.58	0.62			0.25	0.51	0.62	0.39	0.66		
Proportion Explained	0.54	0.19	0.15	0.07	0.05			0.46	0.17	0.17	0.13	0.07			0.38	0.19	0.16	0.22	0.05		
Cumulative Proportion	0.54	0.72	0.87	0.95	1.00			0.46	0.63	0.80	0.93	1.00			0.38	0.78	0.95	0.60	1.00		
RMSR					0.03							0.03							0.03		

Table S3. Exploratory factor analyses of the PANAS

	model	# factors	RMSR	RMSEA	TLI	χ^2 P
0 mg	PANAS_1	2	0.05	0.107	0.847	6.50E-20
	**PANAS_2**	**3**	**0.04**	**0.075**	**0.927**	**1.40E-06**
	PANAS_3	4	0.03	0.066	0.943	0.00029
2 mg	PANAS_1	2	0.05	0.099	0.883	3.10E-16
	**PANAS_2**	**3**	**0.03**	**0.058**	**0.961**	**1.20E-03**
	PANAS_3	4	0.02	0.044	0.980	5.60E-02
4 mg	PANAS_1	2	0.06	0.127	0.821	6.90E-31
	**PANAS_2**	**3**	**0.03**	**0.075**	**0.938**	**1.10E-06**
	PANAS_3	4	0.02	0.048	0.976	2.80E-02

Table S4. Factor loadings of the PANAS

	0 mg gum					2 mg gum					4 mg gum				
**Item**	**Inspired**	**Nervous**	**Attentive**	**h2**	**u2**	**Inspired**	**Nervous**	**Attentive**	**h2**	**u2**	**Inspired**	**Nervous**	**Attentive**	**h2**	**u2**
interested	**0.64**	-0.09	**0.26**	0.48	0.52	**0.68**	-0.12	**0.31**	0.57	0.427	**0.62**	-0.19	**0.40**	0.57	0.43
distressed	0.00	**0.63**	-0.09	0.40	0.60	-0.04	**0.61**	-0.06	0.38	0.617	-0.03	**0.63**	-0.04	0.4	0.6
excited	**0.81**	0.12	0.04	0.68	0.32	**0.79**	0.03	0.14	0.65	0.351	**0.75**	-0.06	0.19	0.59	0.41
strong	**0.71**	-0.07	0.20	0.55	0.45	**0.78**	-0.05	0.10	0.62	0.382	**0.75**	-0.15	0.18	0.62	0.38
enthusiastic	**0.79**	-0.08	0.17	0.66	0.34	**0.87**	-0.12	0.18	0.8	0.201	**0.80**	-0.13	0.29	0.74	0.26
proud	**0.73**	-0.11	0.07	0.55	0.45	**0.79**	0.02	0.03	0.62	0.375	**0.79**	-0.08	0.04	0.63	0.37
irritable	-0.05	**0.54**	-0.17	0.32	0.68	-0.03	**0.57**	-0.09	0.33	0.668	-0.11	**0.60**	-0.23	0.42	0.58
alert	0.47	-0.14	**0.63**	0.63	0.37	**0.59**	-0.02	**0.60**	0.71	0.286	**0.46**	-0.06	**0.78**	0.82	0.18
inspired	**0.86**	0.01	0.13	0.75	0.25	**0.85**	-0.05	0.13	0.74	0.264	**0.85**	-0.09	0.16	0.75	0.25
nervous	-0.05	**0.74**	0.05	0.55	0.45	-0.04	**0.75**	0.11	0.57	0.426	-0.03	**0.82**	-0.03	0.67	0.33
determined	**0.78**	0.04	0.26	0.68	0.33	**0.8**	0.05	0.23	0.69	0.312	**0.81**	0.01	0.23	0.71	0.29
attentive	0.47	-0.10	**0.84**	0.93	0.07	**0.47**	-0.13	**0.82**	0.91	0.092	**0.47**	-0.17	**0.78**	0.86	0.14
jittery	0.01	**0.57**	-0.06	0.33	0.67	0.09	**0.57**	-0.03	0.33	0.672	-0.09	**0.67**	0.05	0.46	0.54
active	**0.74**	0.02	**0.20**	0.59	0.41	**0.74**	0.08	**0.22**	0.6	0.397	**0.67**	0.04	**0.38**	0.59	0.41
afraid	-0.01	**0.59**	0.07	0.35	0.65	-0.02	**0.65**	-0.01	0.42	0.581	-0.04	**0.66**	-0.04	0.44	0.56
SS Loadings	5.07	1.97	1.4			5.56	2.06	1.33			5.04	2.43	1.81		
Proportion Variance	0.34	0.13	0.09			0.37	0.14	0.09			0.34	0.16	0.12		
Cumulative Variance	0.34	0.47	0.56			0.37	0.51	0.6			0.34	0.5	0.62		
Proportion Explained	0.6	0.23	0.17			0.62	0.23	0.15			0.54	0.26	0.2		
Cumulative Proportion	0.6	0.83	1			0.62	0.85	1			0.54	0.8	1		

Table S5. Exploratory factor analyses of the DEN

	model	# factors	RMSR	RMSEA	TLI	BIC	χ^2 P
0 mg	DEN_1	1	0.06	0.122	0.873	-46.99	1.90E-14
	**DEN_2**	**2**	**0.03**	**0.066**	**0.964**	**-90.90**	**5.80E-03**
	DEN_3	3	0.02	0.036	0.990	-73.80	0.2200
2 mg	DEN_1	1	0.08	0.171	0.755	52.74	2.70E-32
	**DEN_2**	**2**	**0.03**	**0.065**	**0.965**	**-90.99**	**5.90E-03**
	DEN_3	3	0.03	0.050	0.98	-69.47	8.80E-02
4 mg	DEN_1	1	0.09	0.195	0.679	116.50	1.90E-44
	**DEN_2**	**2**	**0.04**	**0.075**	**0.953**	**-83.80**	**7.90E-04**
	DEN_3	3	0.04	0.090	0.932	-49.09	2.20E-04

BIC: Bayesian information criterion

Table S6. Factor loadings of the DEN

	0 mg gum				2 mg gum				4 mg gum			
**Item**	**Confused**	**Dizzy**	**h2**	**u2**	**Confused**	**Dizzy**	**h2**	**u2**	**Confused**	**Dizzy**	**h2**	**u2**
Nauseous	**0.59**	0.56	0.66	0.34	**0.60**	0.28	0.44	0.56	**0.62**	0.32	0.48	0.52
Dizzy	0.34	**0.83**	0.79	0.21	0.26	**0.89**	0.85	0.15	0.32	**0.88**	0.88	0.12
Light Headed	0.26	**0.82**	0.74	0.26	0.31	**0.87**	0.85	0.15	0.23	**0.94**	0.93	0.07
Nervous	**0.70**	0.41	0.66	0.34	**0.76**	0.30	0.66	0.34	**0.70**	0.24	0.55	0.45
Sweaty	**0.64**	0.14	0.43	0.57	**0.69**	0.10	0.49	0.51	**0.65**	0.10	0.44	0.57
Headache	**0.54**	0.33	0.39	0.61	**0.58**	0.27	0.41	0.59	**0.54**	0.35	0.42	0.58
Salivation	**0.35**	0.24	0.18	0.82	**0.44**	0.23	0.25	0.75	**0.51**	0.16	0.28	0.72
Heart Pound	**0.57**	0.50	0.58	0.42	**0.67**	0.37	0.59	0.41	**0.59**	0.34	0.46	0.54
Confused	**0.78**	0.30	0.69	0.31	**0.78**	0.29	0.70	0.30	**0.76**	0.29	0.66	0.34
Weak	**0.55**	**0.45**	0.51	0.49	**0.52**	**0.47**	0.49	0.51	**0.55**	**0.48**	0.53	0.47
SS Loadings	3.07	2.57			3.44	2.29			3.23	2.40		
Proportion Variance	0.31	0.26			0.34	0.23			0.32	0.24		
Cumulative Variance	0.31	0.56			0.34	0.57			0.32	0.56		
Proportion Explained	0.54	0.46			0.60	0.40			0.57	0.43		
Cumulative Proportion	0.54	1.00			0.60	1.00			0.57	1.00		
RMSR				0.03				0.03				0.04
